# Clinical evaluation using first-day assessment of patient suitability for successful performance of deep inspiration breath-hold for breast cancer radiotherapy

**DOI:** 10.1186/s13014-026-02919-5

**Published:** 2026-09-01

**Authors:** Bahar Cepni, Vania Batista, Line Hoeltgen, Hanna Waldsperger, Lars Wessel, Jakob Liermann, Semi Harrabi, Fabian Weykamp, Nathalie Arians, Juliane Hörner-Rieber, Jürgen Debus, Eva Meixner

**Affiliations:** 1 Department of Radiation Oncology, Heidelberg University Hospital, Im Neuenheimer Feld 400, 69120 Heidelberg, Germany; 2 Heidelberger Institut für Radioonkologie (HIRO), 69120 Heidelberg, Germany; 3 National Center for Tumor Diseases (NCT), Heidelberg, Germany; 4 Heidelberg Ion Therapy Center (HIT), Im Neuenheimer Feld 450, 69120 Heidelberg, Germany; 5 Clinical Cooperation Unit Radiation Oncology, German Cancer Research Center (DKFZ), Im Neuenheimer Feld 280, 69120 Heidelberg, Germany; 6 Department of Radiation Oncology, University Hospital Düsseldorf, Düsseldorf, Germany

**Keywords:** Breast cancer, Radiotherapy, Deep inspiration breath-hold, DIBH, Cardiac sparing, Patient selection, Comorbidities, Replanning

## Abstract

**Background:**

Deep inspiration breath-hold (DIBH) is a radiotherapy standard technique for cardiac-sparing in breast cancer, particularly for left-sided tumors. However, not all patients can successfully complete DIBH RT in clinical routine, necessitating conversion to free breathing (FB) techniques and replanning. This study aimed to identify clinical factors predictive of DIBH feasibility to optimize patient selection and resource utilization.

**Methods:**

This retrospective study analyzed all patients (*n* = 1920) who underwent curative postoperative breast cancer radiotherapy at our institution between May 2020 and December 2024. All patients underwent initial DIBH feasibility assessment and were evaluated for completion of radiotherapy with DIBH or conversion to FB technique. A detailed subanalysis was performed for all patients treated in 2023 (*n* = 470). Patient-related factors including age, body mass index (BMI), Karnofsky Performance Status (KPS), Charlson Comorbidity Index (CCI), pulmonary comorbidities, smoking status, and geometric surface distance between DIBH and FB planning scans were collected for the subanalysis.

**Results:**

Across the entire cohort (years 2020–2024) of 1,920 patients, 78 replannings (4%) occurred due to conversion from DIBH to free breathing. Increasing age (cut-off of 63 years) was significantly associated with higher replanning rates (*p* = 0.001). In the 2023 subanalysis (*n* = 470), pulmonary comorbidities (*p* = 0.008), higher CCI (*p* = 0.044), and a geometric surface distance of below 1 cm between DIBH and FB CT scans (*p* = 0.0001) were significant predictors of replanning. The Karnofsky Performance Score, BMI, and smoking status showed no significant association with replanning.

**Conclusions:**

Age, pulmonary comorbidities, overall comorbidity burden, and insufficient geometric displacement are significant predictors of DIBH failure in breast cancer radiotherapy. These readily available clinical parameters can optimize patient selection algorithms and inform decisions regarding dual CT simulation workflows. A risk-stratified approach incorporating these factors may reduce unnecessary imaging, decrease planning burden, and improve resource allocation while maintaining optimal cardiac protection for appropriate candidates.

**Clinical trial number:**

The study was registered in the German Clinical Trials Register (DRKS; registration number: DRKS00038550) on 22 December 2025 (https://www.drks.de/).

## Background

Breast cancer is the most common malignant disease in women worldwide, with approximately 2.3 million new cases reported in 2022, representing nearly one in four female cancer diagnoses globally [1]. Postoperative radiotherapy (RT) has been recognized as a standard component of breast cancer treatment, lowering the incidence of local recurrence and increasing overall survival rates [2]. Nevertheless, radiation exposure to the heart during RT for especially left-sided breast cancer has become a significant issue, as epidemiological research indicates that even small doses to the heart are linked to higher long-term cardiovascular disease and death rates [3].

This recognition has driven the adoption of cardiac-sparing techniques, with deep inspiration breath-hold (DIBH) demonstrating superior cardiac protection compared to free breathing (FB) and thus establishing itself as the standard approach for breast radiotherapy, particularly for left-sided tumors due to significantly better cardiac sparing [4, 5]. DIBH exploits the physiological principle that deep inspiration increases lung volume and displaces the heart infero-posteriorly, thereby increasing the distance between the heart and the chest wall [6]. Meta-analyses have demonstrated that deep inspiration breath hold (DIBH) significantly reduces cardiac and pulmonary doses compared with free-breathing (FB) techniques [7, 8]. Especially in left-sided breast cancer, this translates into substantially lower mean heart doses and reduced estimated years of life lost (YLL) due to radiation-induced ischemic heart disease. The benefit appears largely independent of age and is most pronounced in patients with a favourable tumour prognosis, high baseline cardiac risk, or higher mean heart doses under FB, providing a strong rationale for the routine implementation of DIBH in this population [9].

Although it has demonstrated clear dosimetric benefits, there are numerous practical obstacles to the implementation of DIBH. The technique requires patient training, reproducible breath-hold capability (typically > 15 s), specialized monitoring systems, and additional treatment time [10, 11]. A critical workflow consideration is the need to acquire two planning computed tomography (CT) scans per patient—one in FB and one in DIBH—to allow comparative dosimetric evaluation and technique selection. While this dual-scan approach improves treatment planning accuracy, it also increases radiation exposure, patient throughput demands, and planning time, underscoring the importance of effective patient selection strategies.

Emerging evidence suggests that anatomical parameters measured on routine planning CT scans—including total lung volume (TLV), ipsilateral lung volume (ILV), and change in heart height (ΔHH)—can predict DIBH RT dose benefit [12, 13]. Nonetheless, the influence of patient-related factors, especially comorbidities, on assessing the feasibility and successful completion rates of DIBH techniques is still not well defined. While narrative reviews suggest that comorbidities, advanced age, and psychological factors may impede breath-hold capability [14], quantitative data linking specific comorbidities to inability to perform DIBH are sparse.

Given the limited data on patient-specific factors influencing technique selection and the practical implications of dual CT workflows, it is essential to identify patients who both tolerate radiotherapy in DIBH and derive meaningful benefit from it. Therefore, the aim of this study was to evaluate clinical factors assessed during the initial feasibility assessment that predict the successful implementation of DIBH. By identifying these predictors, we seek to support the development of evidence-based, pragmatic selection algorithms for breathing techniques in breast cancer radiotherapy, with the goal of improving resource allocation and avoiding unnecessary dual CT planning in patients unlikely to benefit from or tolerate DIBH.

## Methods

This retrospective study analyzed patients who underwent adjuvant radiotherapy for breast cancer at University Hospital Heidelberg between May 2020 and December 2024. The study was registered at DRKS (registration number: DRKS00038550, registration date 22 December 2025, https://www.drks.de) and approved by the institutional ethics committee (S535/2025) and the requirement for informed consent was waived due to the retrospective nature of the analysis.

Based on institutional guidelines, the suitability of patients for DIBH was assessed via a methodical evaluation procedure with a four-step approach [1]. During the first-day assessment, clinical parameters with patient and tumor characteristics were reviewed and patients were required to exceed a minimum breath-hold duration of 20 s. Patients fulfilling this criterion received structured training supported by written instructional material [2]. A second assessment and training were subsequently performed before the planning CT scan. A DIBH simulation was practiced, and the difference compared to FB was marked on the skin surface at the lateral thorax using a non-permanent marker line. If this difference exceeded 3 mm and the patient was able to follow the breathing commands and maintain an adequate breath hold (> 20 s), an additional DIBH CT scan was performed alongside with the FB CT scan [3]. During the third assessment, treatment planning was conducted, and the difference in planning CT surface distance between FB and DIBH was quantified. For this, the maximum surface distance measured on the axial CT scans at the level of the nipple or at mid-thoracic wall was measured [4]. Radiotherapy was delivered following institutional protocols, with tattoo-based patient positioning and daily surface-guided radiotherapy (SGRT) for DIBH. Patients were treated once daily, five times per week, using either conventionally fractionated or hypofractionated regimens, with volumetric modulated arc therapy (VMAT) or three-dimensional conformal radiotherapy (3D-CRT). Target and organ-at-risk delineation was performed in accordance with ESTRO guidelines [15].

For this study, patient-related factors were retrospectively gathered from medical records and included the age when radiotherapy started (in years), weight (in kg), body mass index (BMI, kg/m²), Karnofsky Performance Status (KPS), and Charlson Comorbidity Index (CCI). The documentation included the existence of chronic, symptomatic, or treatment-requiring lung disease, which was defined as chronic obstructive pulmonary disease (COPD), asthma, pulmonary embolism, or other chronic respiratory diseases requiring active treatment. Smoking habits were classified as current, former, or non-smoker, and pack-years were calculated for current and former smokers. Therapy-related parameters included the target area (chest wall versus breast or implant) and the surgical method (mastectomy versus breast-conserving surgery). Technical parameters included the maximum surface distance difference between FB and DIBH CT scans (cm) as described above.

A subanalysis was performed on a cohort consisting of all patients treated in 2023, which represented the year with the highest number of patients. This cohort was used to assess the predictive value of patient-, tumor-, and therapy-related factors for DIBH feasibility and technique selection.

The primary outcome was the successful completion of radiotherapy with DIBH versus transition to FB technique. Patients were categorized into two groups: the DIBH group, consisting of patients who successfully completed all four steps and received radiotherapy with DIBH technique, and the FB group, consisting of patients who were unable to complete DIBH requirements at any assessment stage and received or converted to radiotherapy with FB technique.

### Statistical analysis

Patient and treatment characteristics between DIBH and FB groups were compared using the Mann-Whitney U tests or Pearson χ2 tests for continuous or categorical data, respectively. A two-sided p-value < 0.05 was considered statistically significant. Receiver operating characteristic (ROC) curve analysis was performed to calculate cut-off age and maximum surface distance values. Age (< 63 vs. ≥63 years) and distance (< 1 cm vs. ≥1 cm) were then dichotomized for further statistical analyses. Statistical analysis was performed using statistical software International Business Machines Corporation (IBM) Statistical Product and Service Solutions (SPSS) (Chicago, Illinois, version 28.0).

## Results

### Overall patient cohort and replanning rates

Between May 2020 and December 2024, 1,920 patients undergoing adjuvant radiotherapy for breast cancer were retrospectively analyzed (2010: *n* = 141, 2021: *n* = 452, 2022: *n* = 425, 2023: *n* = 470, 2024: *n* = 432). Across the entire study period, a total of 119 patients required replanning, involving a second planning CT and a new radiotherapy treatment plan. Replanning that occurred due to anatomical changes (*n* = 24), target volume modifications (*n* = 5), and physics-related treatment planning reasons (*n* = 12) was excluded from further analyses. Replanning due to a conversion from DIBH to FB was performed in 78 patients (4.1%). No statistically significant differences in replanning rates were observed between the treatment years (*p* = 0.377). For the entire cohort (*n* = 1920), a higher age at the start of RT was significantly associated with an increased risk of DIBH to FB replanning (*p* = 0.001). ROC curve analysis identified an age > 63 years as a risk factor for an increased number of conversions from DIBH to FB.

### Subanalysis cohort

**Fig. 1:**
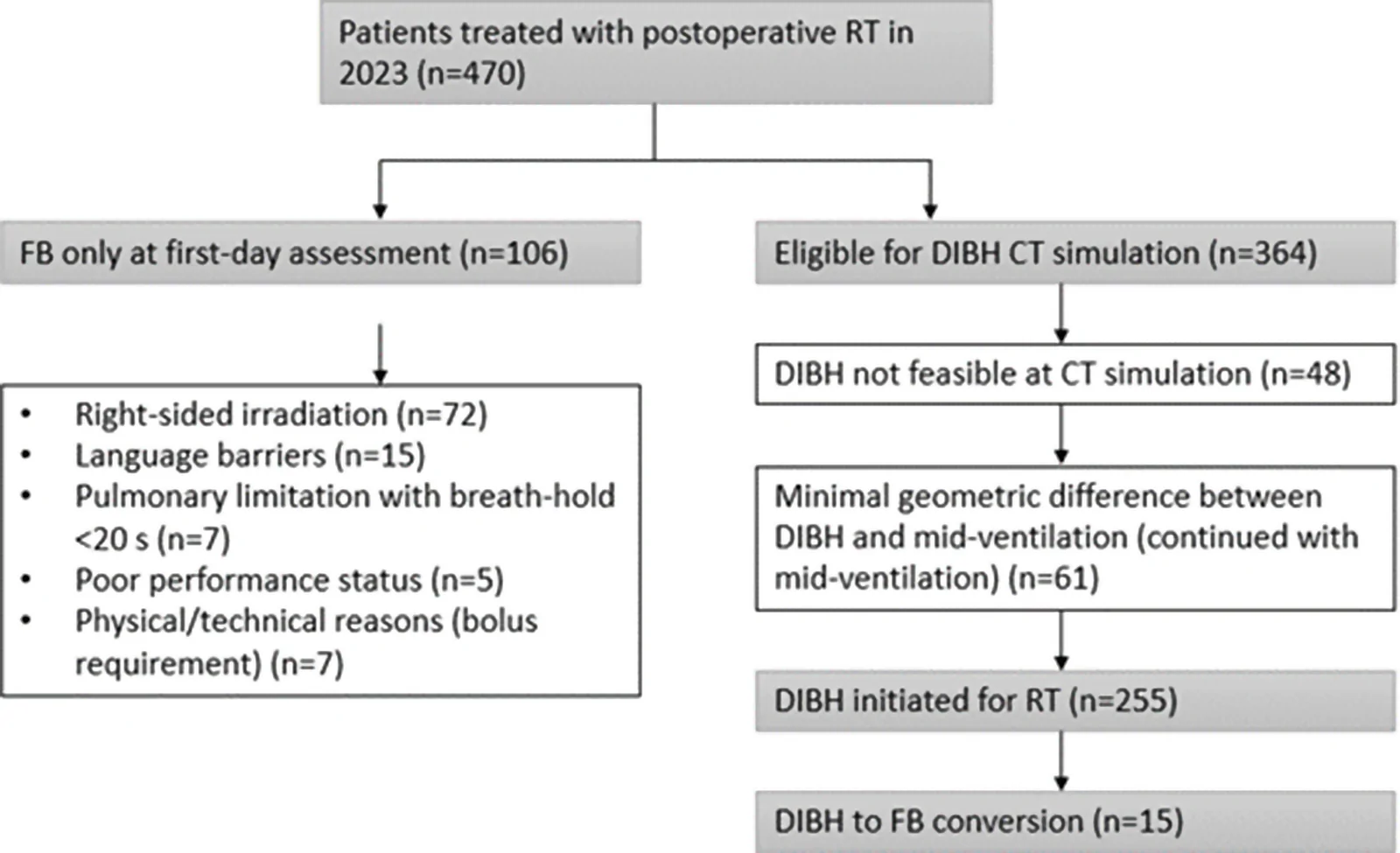
Flow diagram of patient selection and treatment pathways in the 2023 subanalysis cohort. CT: computed tomography, DIBH: deep inspiration breath-hold, FB: free-breathing, RT: Radiotherapy

**Fig. 2:**
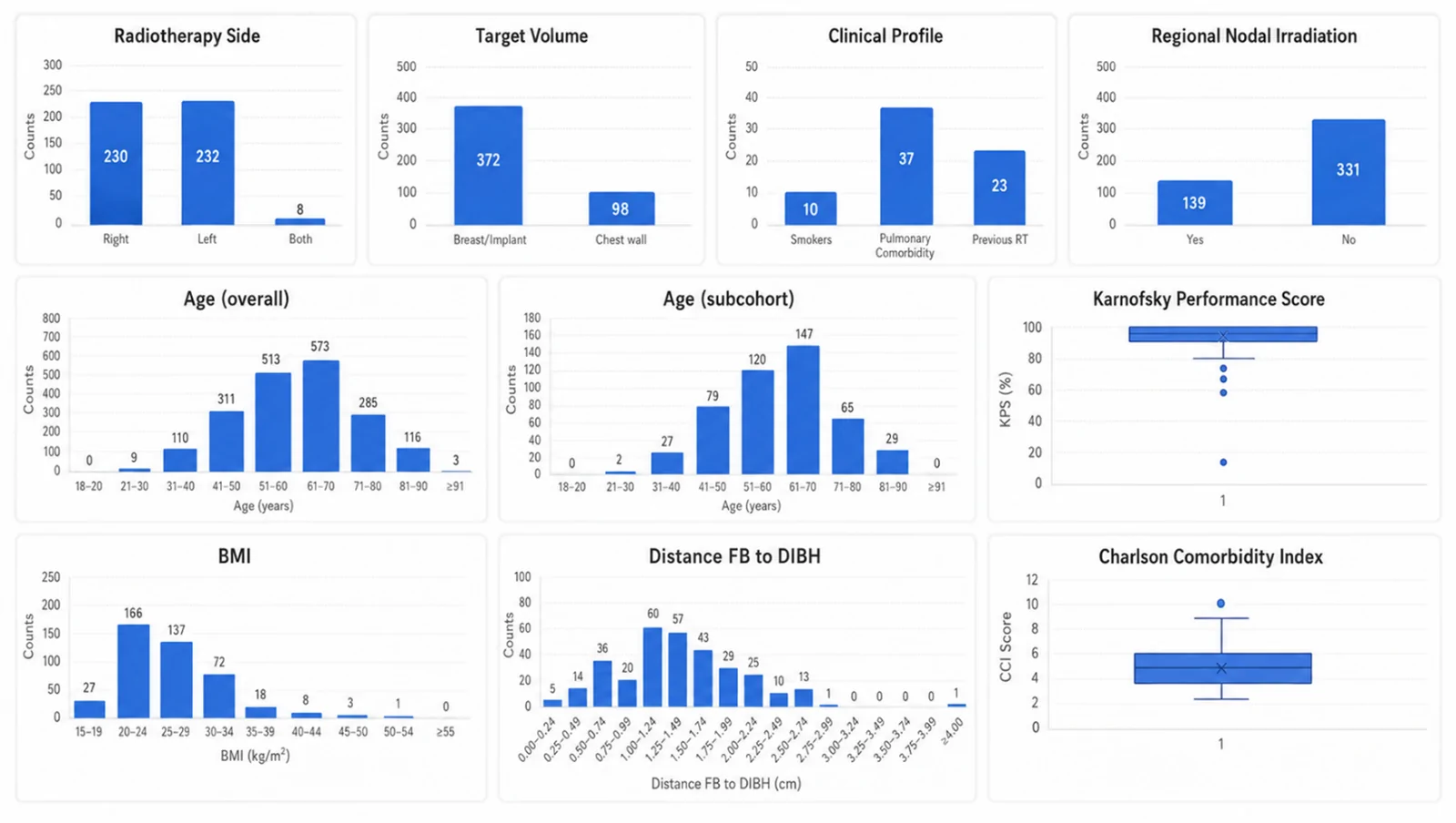
Patient and treatment characteristics of the study cohort. Distributions of radiotherapy side, target volume, clinical profile, regional nodal irradiation, age, BMI, and FB-to-DIBH distance are shown as patient counts. Boxplots depict the distribution of Karnofsky Performance Score (KPS) and Charlson Comorbidity Index (CCI). Age distributions are shown for the overall cohort and the analyzed subcohort

A predefined subanalysis was conducted in patients treated in 2023 (*n* = 470), for whom detailed clinical and technical parameters were available. The median age was 61 years (range: 27–88 years). RT was performed on the left (*n* = 232), right (*n* = 230) or both sides (*n* = 8) and included the chest wall (*n* = 98) or breast/implant (*n* = 372) with (*n* = 139) or without (*n* = 331) regional nodal irradiation. Of the patients who started RT in DIBH technique after clinical and imaging assessments, 94.1% successfully completed the treatment using this method. Fifteen patients (5.9%) converted to FB with median time to replanning or conversion to FB after 2 DIBH fractions (range 0–12 fractions). An overview of patient selection and treatment technique assignment is shown in Fig. 1. Baseline patient and treatment characteristics of the study cohort are presented in Table 1 and Fig. 2.

**Table 1:** Patient and treatment characteristics

Variable	Value
Age, years	61 (27–88)
RT side	
Right	230 (49.0%)
Left	232 (49.4%)
Both	8 (1.7%)
Target volume	
Breast/implant	372 (79.1%)
Chest wall	98 (20.9%)
Regional nodal irradiation	
Yes	139 (29.6%)
No	331 (70.4%)
KPS	90 (50–100)
CCI	4 (2–9)
Smokers	10 (2.1%)
Pack years	0 (0–60)
Pulmonary comorbidity	37 (7.9%)
Distance FB to DIBH, cm	1.3 (0.1–4.0)

Data are presented as median (range) or n (%), unless otherwise specified

† Percentages are calculated based on the entire cohort (*n* = 470). Smoking status was available for 320 patients, and pulmonary comorbidity data were available for 451 patients. Missing data were not imputed

**Fig. 3:**
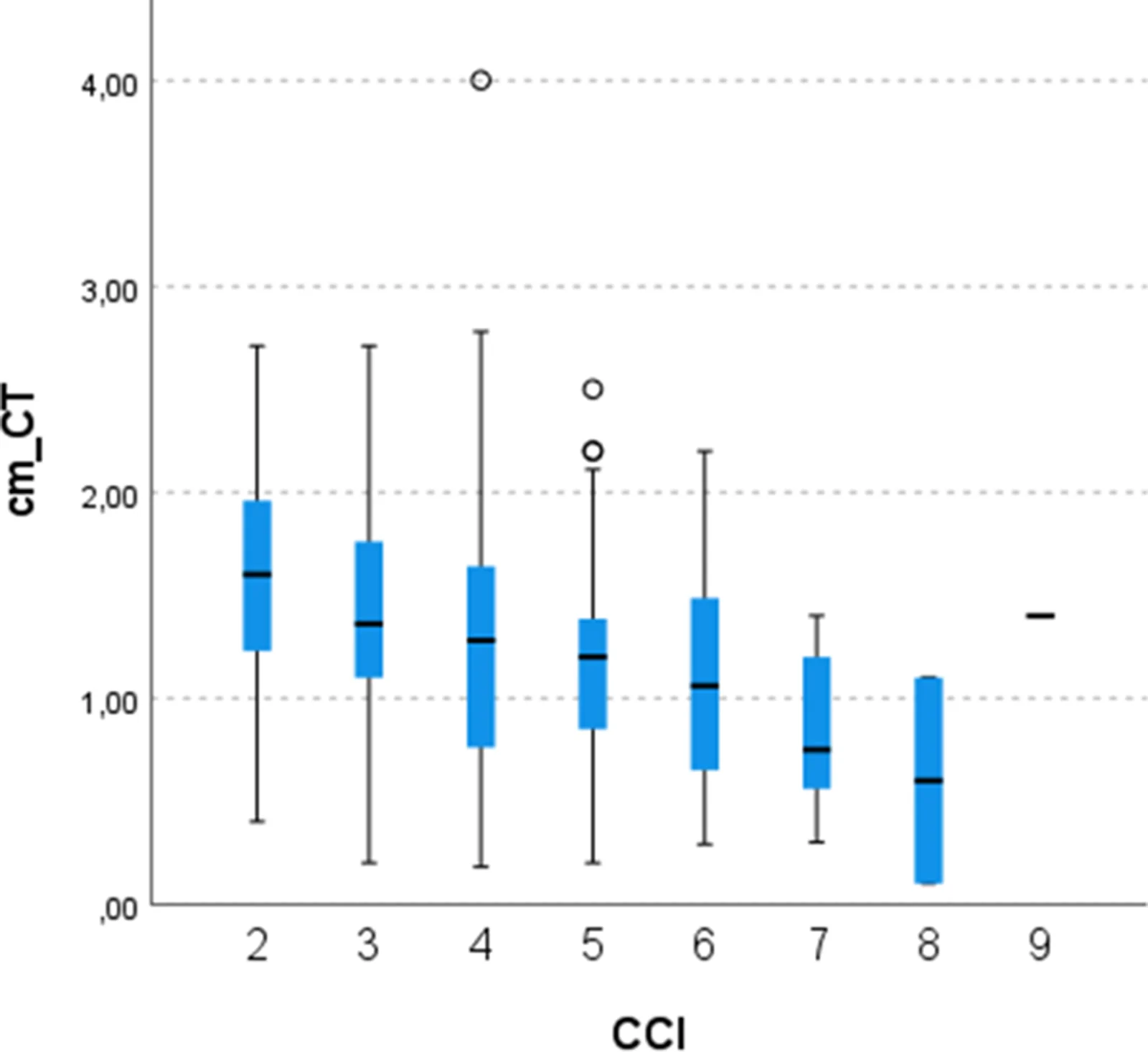
Correlation between Charlson Comorbidity Index (CCI) and the geometric distance between free breathing (FB) and deep inspiration breath hold (DIBH), measured in centimeters. Boxplots with minimum, maximum and median values. °: outlier

The presence of a pulmonary comorbidity (assessed in *n* = 451) was significantly associated with the risk of DIBH to FB replanning (*p* = 0.008). Likewise, comorbidity burden, assessed using the Charlson Comorbidity Index (*n* = 458), was significantly linked to replanning (*p* = 0.044). A higher CCI, representing a large number of comorbidities, showed a significantly (*p* = 0.018) smaller maximum centimeter distance between FB and DIBH CTs (Fig. 3).

**Fig. 4:**
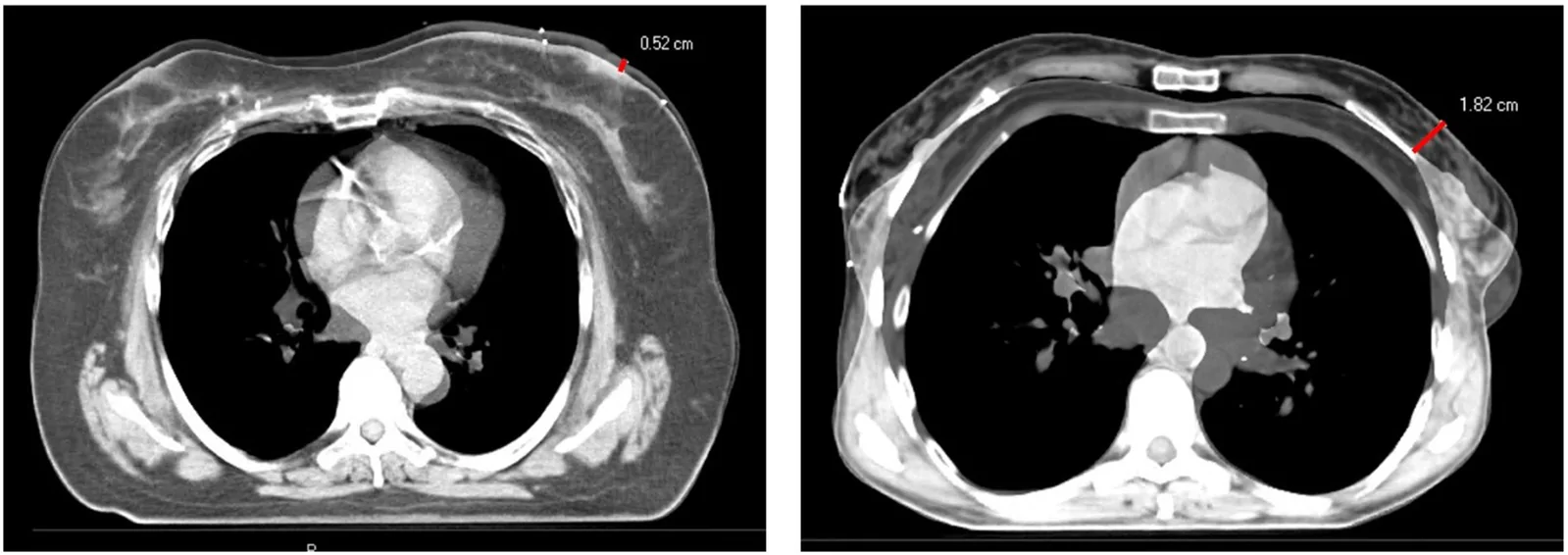
Representative example of the distance measurement between free breathing (FB) CT and deep inspiration breath hold (DIBH) CT

Additionally, insufficient geometric differences between FB and DIBH CT scans, defined by the maximum difference in centimeters, was a significant factor for replanning (*p* = 0.018). A representative example illustrating the method used to measure the geometric distance between FB CT and DIBH CT scans is provided in Fig. 4. ROC curve analysis identified a distance below 1 cm as a risk factor for an increased number of conversions from DIBH to FB.

Performance status, assessed using the Karnofsky index (*n* = 448, *p* = 0.891), was not significantly associated with replanning. Similarly, the body mass index (BMI) (*n* = 432, *p* = 0.324), the smoking status (*n* = 320, *p* = 0.688) and pack years (*p* = 0.169) showed no significant association with replanning. Being a non-smoker, previous irradiation of the contralateral breast, the presence of language barriers, lymph node irradiation, and treatment of the breast or chest wall, with or without irradiation of the lymphatic drainage pathways, were not significantly associated with replanning.

## Discussion

This retrospective analysis of 1,920 patients undergoing adjuvant breast radiotherapy identified several patient-related factors associated with conversion from DIBH to a FB technique. Across the entire cohort (2020–2024), increasing age was significantly linked to higher replanning rates. In the detailed 2023 subanalysis, pulmonary comorbidities, a higher Charlson Comorbidity Index (CCI), and insufficient geometric differences between FB and DIBH CT scans emerged as significant predictors of technique conversion. Conversely, factors such as the performance status, BMI, and smoking status did not show a significant correlation with replanning.

Age was a consistent predictor of replanning in the overall cohort, with older patients more frequently transitioning from DIBH to FB. This finding aligns with new predictive models that incorporate age alongside anatomical or functional parameters when determining eligible patients for DIBH. Prior studies by Sun et al. and Linge et al. identified age as an independent predictor of DIBH-related cardiac dose reduction or technique suitability, particularly in left-sided breast cancer [16, 17].

The relationship between age and the feasibility of DIBH is probably influenced by multiple factors. With age, there is often a reduction in pulmonary capabilities, characterized by lower vital capacity and inspiratory reserve, which may limit lung expansion during breath retention. Prior studies provide evidence for this mechanistic relationship, highlighting strong ties between inspiratory capacity and reductions in cardiac dose [8]. Moreover, the burden of comorbid conditions related to age, diminished physical endurance, and cognitive elements influencing adherence to instructions may further hinder prolonged breath-holding abilities. Nevertheless, chronological age alone is likely an imperfect surrogate for physiological reserve and should be interpreted in conjunction with functional and comorbidity assessments.

Pulmonary comorbidities were a strong predictor of replanning in the 2023 subanalysis, providing quantitative confirmation of a clinically intuitive relationship. Patients with chronic or symptomatic pulmonary disease demonstrated significantly higher rates of DIBH failure, consistent with current literature showing that achievable lung expansion is a key determinant of heart-sparing benefit [18].

The importance of pulmonary function is well established. Gaál et al. indicated that modifications in lung volume during free breathing and DIBH significantly predict reductions in heart dose, with spirometric factors like vital capacity being strongly linked to the expected inhalation expansion realized [18]. Furthermore, research on real-time surface tracking has indicated that individuals with cardiopulmonary disorders exhibit reduced consistency in breath-holding, although structured training programs have been shown to significantly improve DIBH feasibility and reproducibility [19]. In our cohort, patients received two structured oral training sessions (at the first and second assessment) in addition to written instructions and informational flyers to support DIBH performance.

Beyond pulmonary disease alone, the CCI emerged as a novel and significant predictor of replanning. To our knowledge, this is the first study to demonstrate a quantitative association between a standardized comorbidity index and DIBH feasibility. The predictive value of the CCI likely reflects cumulative physiological burden, reduced cardiopulmonary reserve, and diminished tolerance for the physical and cognitive demands of repeated breath-holds. Importantly, the CCI is routinely available in oncology practice, making it an attractive candidate for incorporation into DIBH selection algorithms.

Insufficient geometric difference between FB and DIBH CT scans was also significantly associated with replanning, reinforcing the concept that not all patients derive meaningful benefit from DIBH. This finding is consistent with prior studies proposing anatomical thresholds—such as heart-in-field distance or baseline heart dose—to guide patient selection [20, 21].

While proposed thresholds vary widely and remain incompletely validated, the present study demonstrates that simple geometric metrics used in routine planning can meaningfully inform technique selection.

Several commonly considered clinical factors did not significantly predict replanning. The performance status, evaluated using the Karnofsky score, demonstrated no correlation, probably indicating limited variability within a population aimed at curative treatment. In contrast to previous reports identifying BMI as a significant predictor of DIBH feasibility and dosimetric outcomes [22], BMI did not predict replanning in our cohort, suggesting that the influence of body habitus on lung mechanics and cardiac displacement may be more complex and potentially counterbalanced by competing physiological effects. Smoking status alone was not predictive, likely because its impact on pulmonary function is better captured by the presence of established pulmonary disease rather than smoking history per se.

These findings have important implications for clinical workflow optimization. Routine acquisition of dual CT scans for all patients considered for DIBH represents a substantial resource burden. In the present cohort, a subset of patients ultimately received RT in free breathing due to DIBH infeasibility or limited benefit. Recognizing patients who are unlikely to benefit from DIBH at an early stage may help minimize unnecessary imaging, lessen planning demands, and alleviate patient stress.

A risk-stratified approach incorporating age, pulmonary comorbidities, CCI, and simple geometric parameters may help guide decisions regarding dual-scan acquisition, training intensity, or alternative heart-sparing techniques. Such an approach aligns with previously proposed decision models and could be implemented using readily available clinical data [7, 17, 18]. Prospective validation of any selection algorithm, however, is essential before routine clinical adoption.

Our findings on patient-related predictors of DIBH feasibility highlight the heterogeneity in who can successfully perform DIBH. These results align with recent modeling studies in Belgium, which demonstrated that DIBH was cost-effective in approximately 58% of patients, with the greatest benefit observed in those with left-sided disease, nodal involvement, elevated cardiovascular risk, smoking history, or older age. Universal implementation of DIBH reduced maximum radiotherapy throughput by 21.7%, whereas a targeted, risk-adapted approach reduced throughput by only 13.1%, emphasizing the operational advantages of selective allocation [23]. Another Belgian study corroborated these findings, showing that DIBH reduced total cardiovascular events by 1.72% and fatal events by 0.69% over 20 years, providing an incremental 0.04 QALYs per patient. While treatment times increased and costs rose by €617 per patient, the incremental cost-effectiveness ratio remained well below conventional thresholds (€14,023/QALY), confirming that DIBH is economically favorable despite workflow implications [24].

Furthermore, the latest German S3 guideline for breast cancer explicitly recommends the use of DIBH or respiratory-gated techniques for left-sided radiotherapy to reduce cardiac exposure [25]. Together, these data support a risk-stratified, value-based approach to DIBH allocation: prioritizing patients most likely to benefit can optimize both clinical outcomes and resource utilization, while aligning with current guideline recommendations and evidence-based best practices.

Ultimately, evaluating the rates of DIBH failure or the need for replanning across different studies proves difficult because of varied definitions, selection standards, and monitoring techniques. The pragmatic definition of replanning used in the present study—conversion from DIBH to FB for any reason—reflects real-world clinical practice, but does not distinguish between patient-related and dosimetric causes. Future studies should standardize reporting and disentangle these mechanisms to further refine patient selection strategies.

Several research priorities emerge from this study and the existing DIBH literature. First, prospective external validation of clinical prediction models incorporating age, comorbidity burden, pulmonary disease, and geometric parameters is needed, using standardized definitions of DIBH success and failure and clinically relevant subgroups. Second, disease-specific analyses of pulmonary comorbidities are warranted, as quantitative data differentiating between COPD, asthma, and interstitial lung disease remain limited. Thirds, cost-effectiveness analyses comparing dual-CT workflows with alternative preselection or triage strategies are needed to optimize resource utilization. Finally, long-term outcome studies are essential to determine whether DIBH-related cardiac dose reductions translate into clinically meaningful reductions in cardiovascular morbidity.

The present study has several limitations. The retrospective design limits causal inference and introduces potential selection bias, as clinical decisions about DIBH candidacy were made by treating physicians based on individual patient assessment rather than standardized research criteria. Pulmonary comorbidity was defined heterogeneously and failed to differentiate between specific diagnoses, disease severity, or treatment status. Systematic collection of objective pulmonary function data (spirometry) was lacking, hindering a direct evaluation of the relationship between pulmonary function and the feasibility of DIBH. The cohort from the 2023 subanalysis is derived from a single institution and may not be representative of other populations, especially those with varying demographic features, comorbidity patterns, or treatment methods. Factors reported by patients, including anxiety, claustrophobia, perceived dyspnea, and motivation, were not assessed in a systematic manner, though they could affect DIBH tolerance independently of objective physiological measures [6].

However, our results comprise an extensive participant cohort recruited within a short period of time that offers strong statistical strength for identifying correlations and represents authentic clinical practice trends. The detailed 2023 subanalysis cohort included comprehensive clinical data on performance status, comorbidities, smoking history, and technical parameters that are often missing from retrospective studies. The use of the validated Charlson Comorbidity Index represents a methodological strength and enables comparison with broader oncology literature. The pragmatic definition of replanning as the primary outcome reflects clinically relevant decision-making and resource utilization. The study was conducted at a high-volume academic center with standardized DIBH protocols and experienced personnel, ensuring technical consistency.

## Conclusions

In conclusion, this large retrospective analysis identifies age, pulmonary comorbidities, comorbidity burden, and limited geometric displacement as key predictors of DIBH failure in postoperative breast cancer radiotherapy. These readily available clinical parameters at first-day assessment can support a risk-stratified approach to patient selection, potentially reducing unnecessary dual-CT acquisition and optimizing treatment workflows. Incorporating comorbidity assessment and simple geometric measures into clinical decision-making may improve the efficiency and targeting of cardiac-sparing strategies. Prospective validation and long-term outcome studies are warranted to confirm the clinical impact of this approach.

## Data Availability

The datasets used and/or analysed during the current study are available from the corresponding author on reasonable request.

## Abbreviations

DIBH: Deep inspiration breath–hold
RT: Radiotherapy
FB: Free breathing
BMI: Body mass index
KPS: Karnofsky Performance Status
CCI: Charlson Comorbidity Index
CT: Computed tomography
TLV: Total lung volume
ILV: Ipsilateral lung volume
ΔHH: Change in heart height
DRKS: Deutsches Register Klinischer Studien (German Clinical Trials Register)
VMAT: Volumetric modulated arc therapy
3D–CRT: Three–dimensional conformal radiotherapy
ESTRO: European Society for Radiotherapy and Oncology
SGRT: Surface–guided radiotherapy
ROC: Receiver operating characteristic
IBM: International Business Machines Corporation
SPSS: Statistical Package for the Social Sciences
COPD: Chronic obstructive pulmonary disease

## References

[1] Kim J, Harper A, McCormack V, Sung H, Houssami N, Morgan E, et al. Global patterns and trends in breast cancer incidence and mortality across 185 countries. Nat Med. 2025;31(4):1154–62. 39994475 10.1038/s41591-025-03502-3

[2] Darby S, McGale P, Correa C, Taylor C, Arriagada R, Clarke M, et al. Effect of radiotherapy after breast-conserving surgery on 10-year recurrence and 15-year breast cancer death: meta-analysis of individual patient data for 10,801 women in 17 randomised trials. Lancet. 2011;378(9804):1707–16. 22019144 10.1016/S0140-6736(11)61629-2 PMC3254252

[3] Darby SC, Ewertz M, McGale P, Bennet AM, Blom-Goldman U, Brønnum D, et al. Risk of ischemic heart disease in women after radiotherapy for breast cancer. N Engl J Med. 2013;368(11):987–98. 23484825 10.1056/NEJMoa1209825

[4] Bergom C, Currey A, Desai N, Tai A, Strauss JB. Deep Inspiration Breath Hold: Techniques and Advantages for Cardiac Sparing During Breast Cancer Irradiation. Front Oncol. 2018;8:87. 29670854 10.3389/fonc.2018.00087 PMC5893752

[5] Yeung R, Conroy L, Long K, Walrath D, Li H, Smith W, et al. Cardiac dose reduction with deep inspiration breath hold for left-sided breast cancer radiotherapy patients with and without regional nodal irradiation. Radiat Oncol. 2015;10:200. 26391237 10.1186/s13014-015-0511-8 PMC4578779

[6] Rochet N, Drake JI, Harrington K, Wolfgang JA, Napolitano B, Sadek BT, et al. Deep inspiration breath-hold technique in left-sided breast cancer radiation therapy: Evaluating cardiac contact distance as a predictor of cardiac exposure for patient selection. Pract Radiat Oncol. 2015;5(3):e127–34. 25413399 10.1016/j.prro.2014.08.003

[7] Schröder C, Kirschke S, Blank E, Rohrberg S, Förster R, Buchali A. Deep inspiration breath-hold for patients with left-sided breast cancer - A one-fits-all approach? A prospective analysis of patient selection using dosimetrical and practical aspects. Br J Radiol. 2022;95(1135):20210295. 34111954 10.1259/bjr.20210295 PMC10996328

[8] Yamauchi R, Mizuno N, Itazawa T, Saitoh H, Kawamori J. Dosimetric evaluation of deep inspiration breath hold for left-sided breast cancer: analysis of patient-specific parameters related to heart dose reduction. J Radiat Res. 2020;61(3):447–56. 32100831 10.1093/jrr/rraa006 PMC7299269

[9] Simonetto C, Eidemüller M, Gaasch A, Pazos M, Schönecker S, Reitz D, et al. Does deep inspiration breath-hold prolong life? Individual risk estimates of ischaemic heart disease after breast cancer radiotherapy. Radiother Oncol. 2019;131:202–7. 30097250 10.1016/j.radonc.2018.07.024

[10] Bruzzaniti V, Abate A, Pinnarò P, D’Andrea M, Infusino E, Landoni V, et al. Dosimetric and clinical advantages of deep inspiration breath-hold (DIBH) during radiotherapy of breast cancer. J Exp Clin Cancer Res. 2013;32(1):88. 24423396 10.1186/1756-9966-32-88 PMC3826503

[11] Mondal M, Mallik S, Goswami J. Deep inspiratory breath-hold technique for patients with left-sided breast cancer in resource-poor settings: Are there any patient selection criteria? Cancer Res Stat Treat. 2023;6(3).

[12] Zhu H, Sun H, Zhang J, Xie Y, Jassem J, Franco P, et al. Cardiac exposure in left-sided breast cancer patients undergoing deep inspiratory breath hold radiation therapy. Gland Surg. 2023;12(5):677–86. 37284707 10.21037/gs-23-160 PMC10240437

[13] Hayden AJ, Rains M, Tiver K. Deep inspiration breath hold technique reduces heart dose from radiotherapy for left-sided breast cancer. J Med Imaging Radiat Oncol. 2012;56(4):464–72. 22883657 10.1111/j.1754-9485.2012.02405.x

[14] Hanczyk E, Piecuch D, Kopcial S, Jonska-Gmyrek J. Factors Affecting the Effectiveness of DIBH (Deep Inspiratory Breath Hold) in Patients with Left Breast Cancer: A Narrative Review. Appl Sci [Internet]. 2024;14(16):7287.

[15] Offersen BV, Boersma LJ, Kirkove C, Hol S, Aznar MC, Biete Sola A, et al. ESTRO consensus guideline on target volume delineation for elective radiation therapy of early stage breast cancer. Radiother Oncol. 2015;114(1):3–10. 25630428 10.1016/j.radonc.2014.11.030

[16] Sun X, Zhang Y, Wu XM, Zhou Y. Prediction models of cardiac sparing during deep inspiration breath-hold in left-sided breast cancer with internal mammary node irradiation. Int J Radiation Oncol Biol Phys. 2024.

[17] Linge A, Ketzmerick C, Gurtner K, Hoinkis C, Löck S, Krause M. Development of a model to guide the decision for radiotherapy in deep-inspiration breath-hold in patients with left-sided breast cancer. Strahlentherapie und Onkologie: Organ der Deutschen Rontgengesellschaft [et al]; 2025. 10.1007/s00066-025-02438-4 PMC12819490 40728585

[18] Gaál S, Kahán Z, Paczona V, Kószó R, Drencsényi R, Szabó J, et al. Deep-inspirational breath-hold (DIBH) technique in left-sided breast cancer: various aspects of clinical utility. Radiat Oncol. 2021;16. 10.1186/s13014-021-01816-3 PMC8117634 33985547

[19] Kefeli AU, Diremsizoglu U, Erdogan S, Karabey AU, Konuk AO, Tirpanci B, et al. Patient coaching for deep inspiration breath hold decreases set-up duration and left anterior descending artery dose for left-sided breast cancer radiotherapy. Support Care Cancer. 2025;33(5):387. 40240656 10.1007/s00520-025-09446-1 PMC12003517

[20] Ferdinand S, Mondal M, Mallik S, Goswami J, Das S, Manir KS, et al. Dosimetric analysis of Deep Inspiratory Breath-hold technique (DIBH) in left-sided breast cancer radiotherapy and evaluation of pre-treatment predictors of cardiac doses for guiding patient selection for DIBH. Tech Innov Patient Support Radiat Oncol. 2021;17:25–31. 33681484 10.1016/j.tipsro.2021.02.006 PMC7930610

[21] Zhou Y, Xu J, Xu F, Li Y, Li H, Pan L, et al. Selection criteria and method for deep inspiration breath-hold in patients with left breast cancer undergoing PMRT/IMRT. Clin Translational Radiation Oncol. 2024;48:100812. 10.1016/j.ctro.2024.100812 PMC11263495 39044781

[22] Koide Y, Shimizu H, Aoyama T, Kitagawa T, Miyauchi R, Watanabe Y, et al. Preoperative spirometry and BMI in deep inspiration breath-hold radiotherapy: the early detection of cardiac and lung dose predictors without radiation exposure. Radiat Oncol. 2022;17(1):35. 35183194 10.1186/s13014-022-02002-9 PMC8858484

[23] Busschaert SL, Kimpe E, Barbé K, Gevaert T, De Ridder M, Putman K. Deep inspiration breath-hold for breast cancer radiotherapy: a modelling study of targeted versus non-targeted strategy for clinical and operational optimisation. Lancet Reg Health – Europe. 2026;60. 10.1016/j.lanepe.2025.101509 PMC12617637 41245119

[24] Busschaert S-L, Kimpe E, Gevaert T, De Ridder M, Putman K. Deep Inspiration Breath Hold in Left-Sided Breast Radiotherapy: A Balance Between Side Effects and Costs. JACC: CardioOncology. 2024;6(4):514–25. 39239337 10.1016/j.jaccao.2024.04.009 PMC11372305

[25] Leitlinienprogramm O, Früherkennung. Diagnostik, Therapie und Nachsorge des Mammakarzinoms – Langversion 5.0. Berlin: Leitlinienprogramm Onkologie; 2025.

